# Supplementary material for: Maturation of the Human Immunoglobulin Heavy Chain Repertoire With Age
Source: Front Immunol. 2020 Aug 6;11:1734. doi: 10.3389/fimmu.2020.01734 (PMC7424015; doi:10.3389/fimmu.2020.01734)
Supplement: Supplementary file 1 [file Data_Sheet_1.docx]

**Supplementary information**


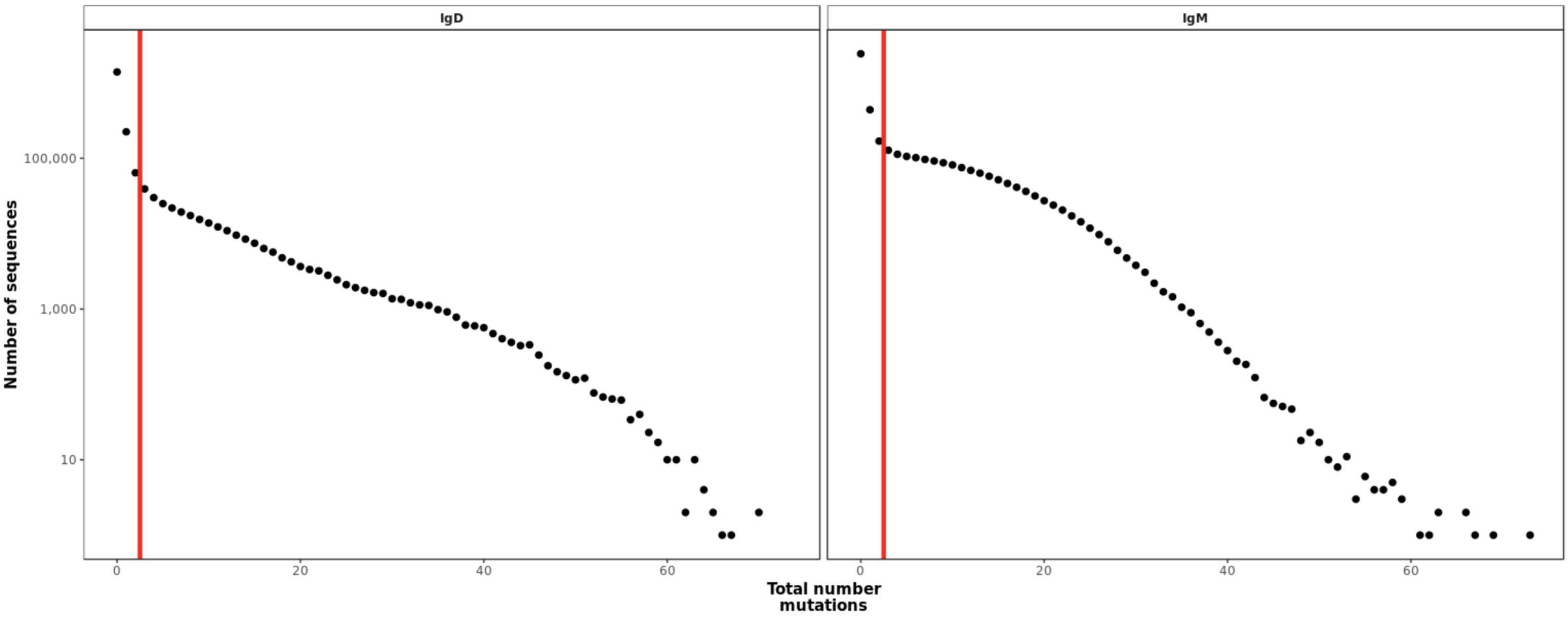


**Supplementary figure 1:** Distribution of somatic hypermutation in all IgD and IgM transcripts. The vertical line indicates the threshold chosen (mutation n between 2 and 3) to separate naïve and memory repertoires for IgM and IgD sequences.

**
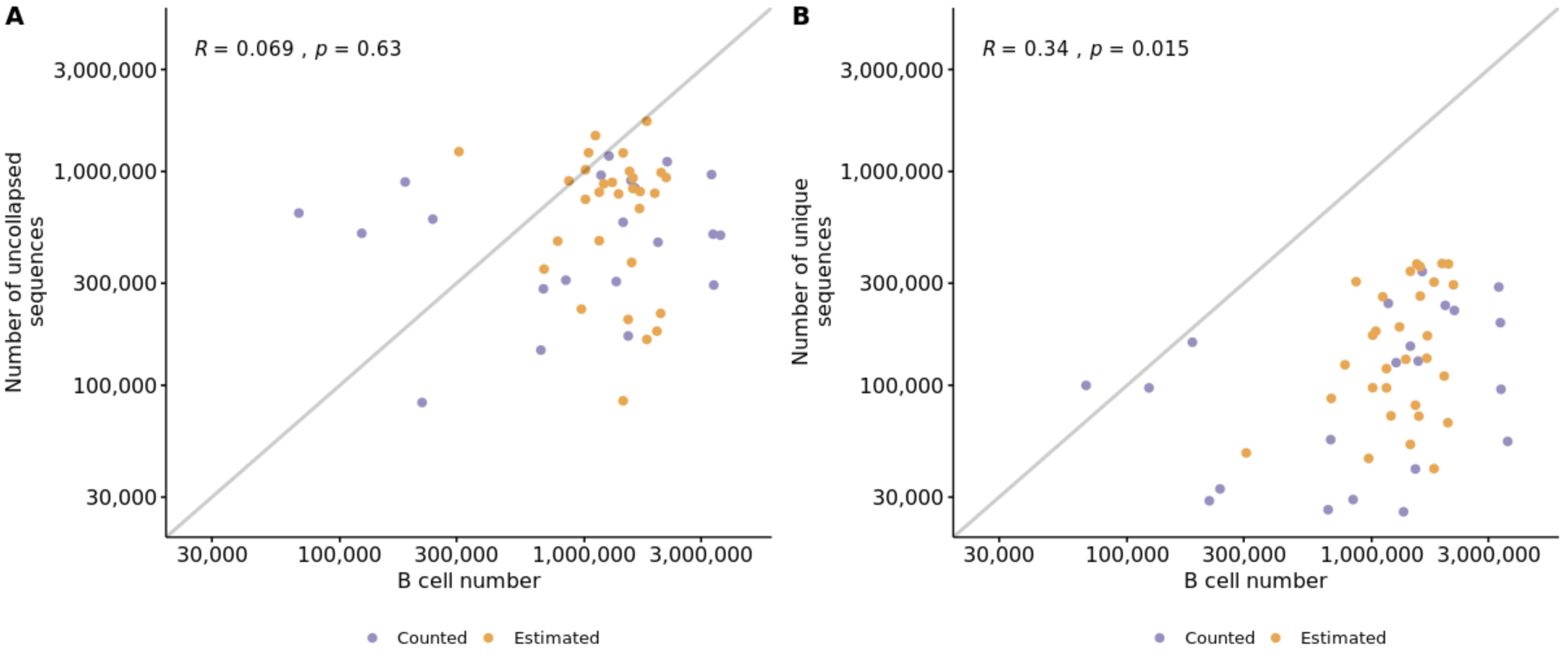
**

**Supplementary figure 2:** Correlation between cell number in a sample, and the number of sequences for that sample *A* before and *B* after collapsing (Pearson correlation coefficient). The B-cell number was either based on actual counts or estimated using PBMC counts and the median percentage of age-dependent reference values.

**
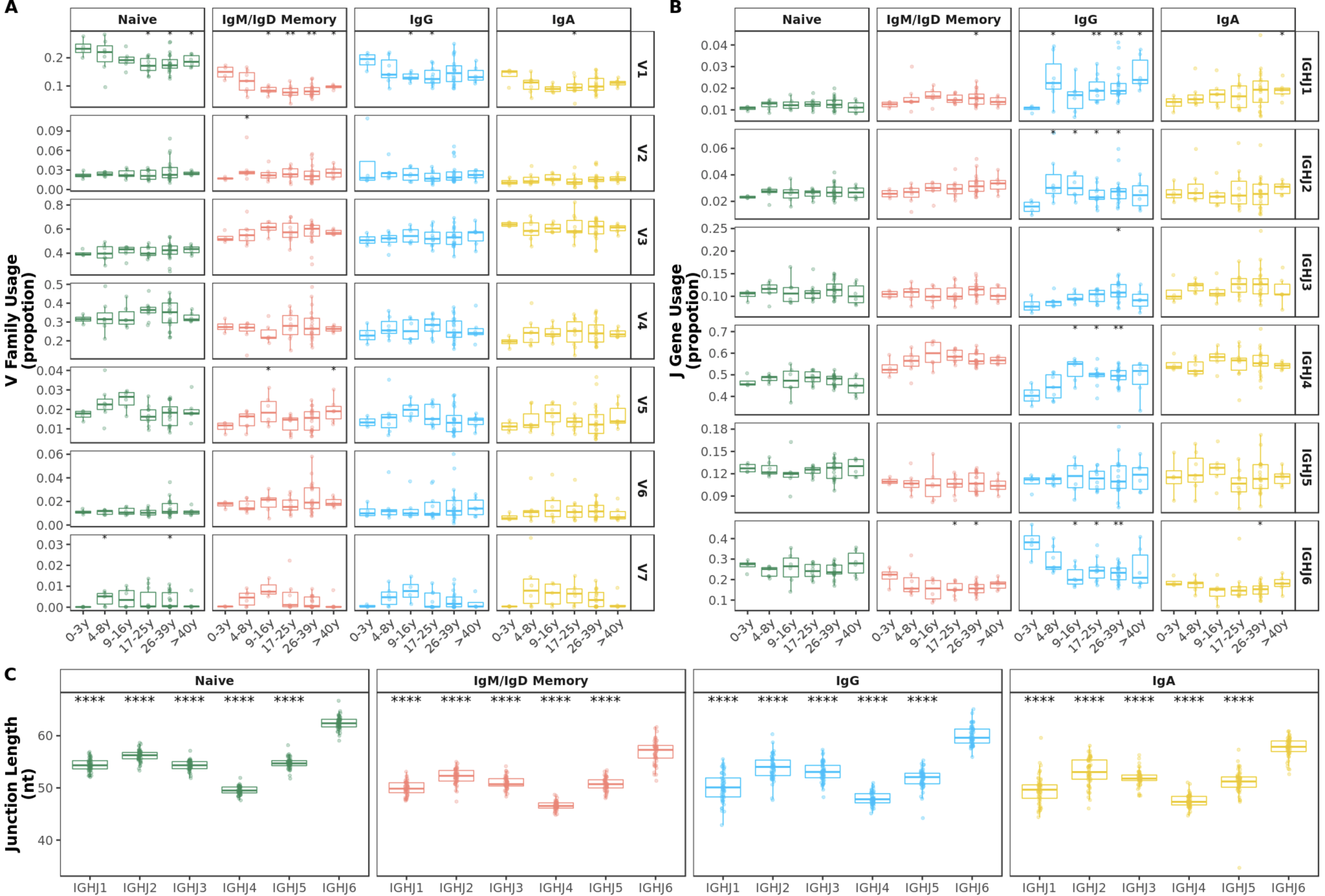
**

**Supplementary figure 3:** *A* V family and *B* J gene usage by age band. Comparison of each age group to the 0-3y group was performed using the Wilcoxon test. *C* IGHJ6 transcripts show significantly longer junctions. Comparison of each gene to IGHJ6 was performed using the Wilcoxon test. *p<0.05, **p<0.01, ***p<0.001, ****p<0.0001

**
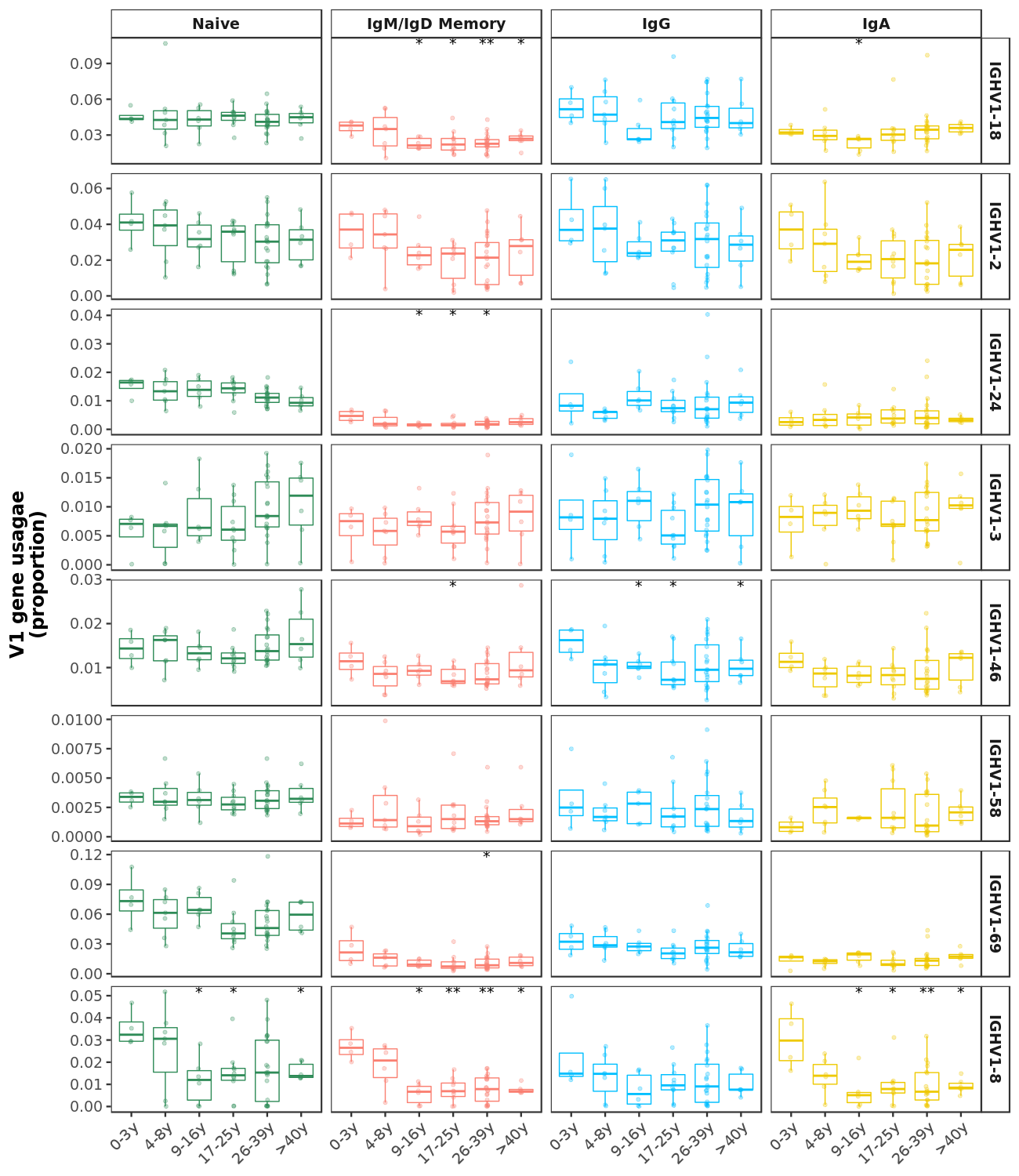
**

**Supplementary figure 4:** Proportion of the top 8 V1 family genes out of all sequences by age band. The decrease seen in V1 family usage is a result of a decrease in multiple individual genes. Comparison of each age group to the 0-3y group was performed using the Wilcoxon test. *p<0.05, **p<0.01

**
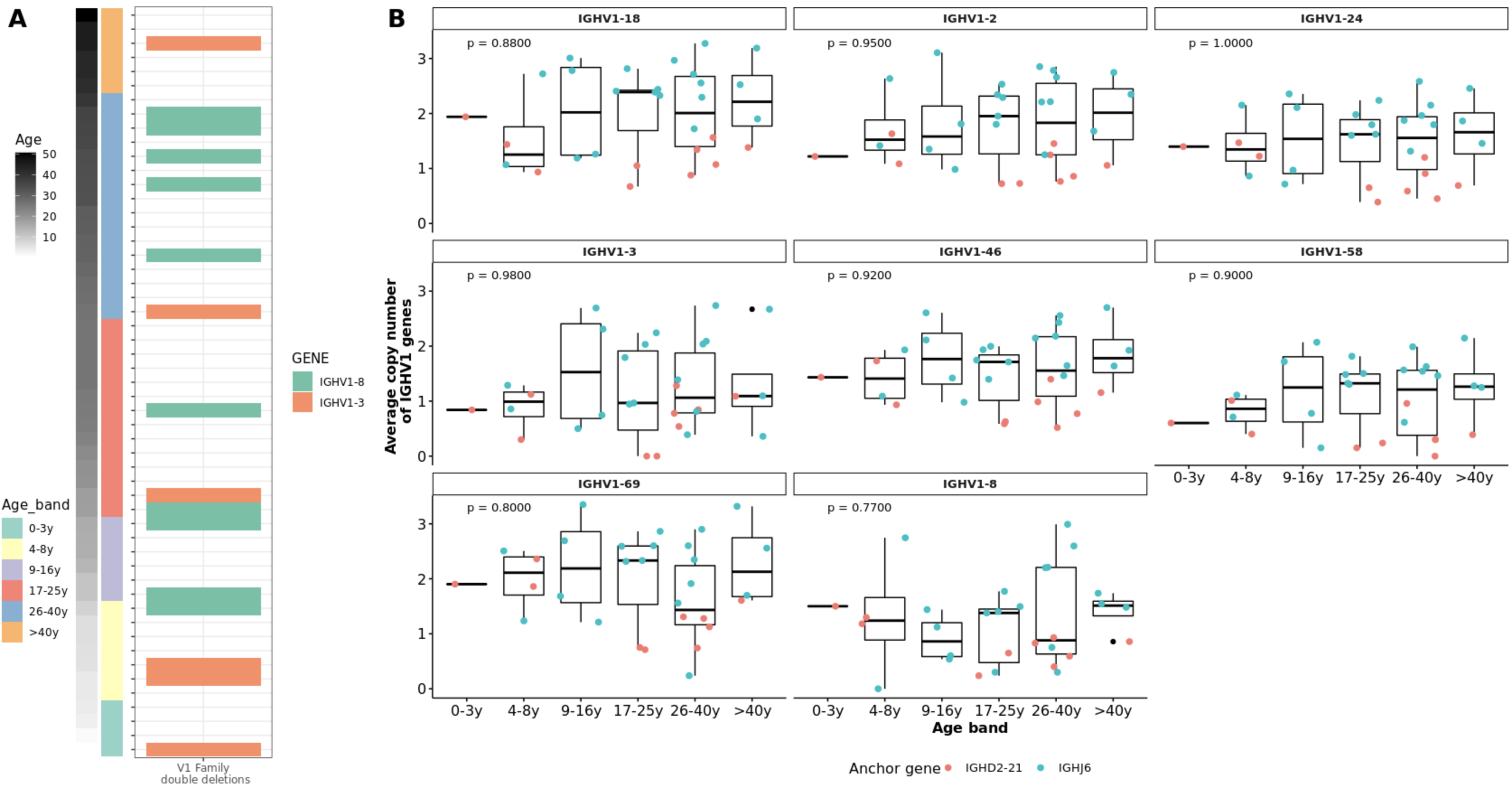
**

**Supplementary figure 5:** *A* Detected double chromosome deletion of V1 family genes by individual (row). Individuals are ordered by age. *B* Average number of copies of V1 genes does not correlate with age. Statistical differences between groups were tested using the Kruskal-Wallis test.


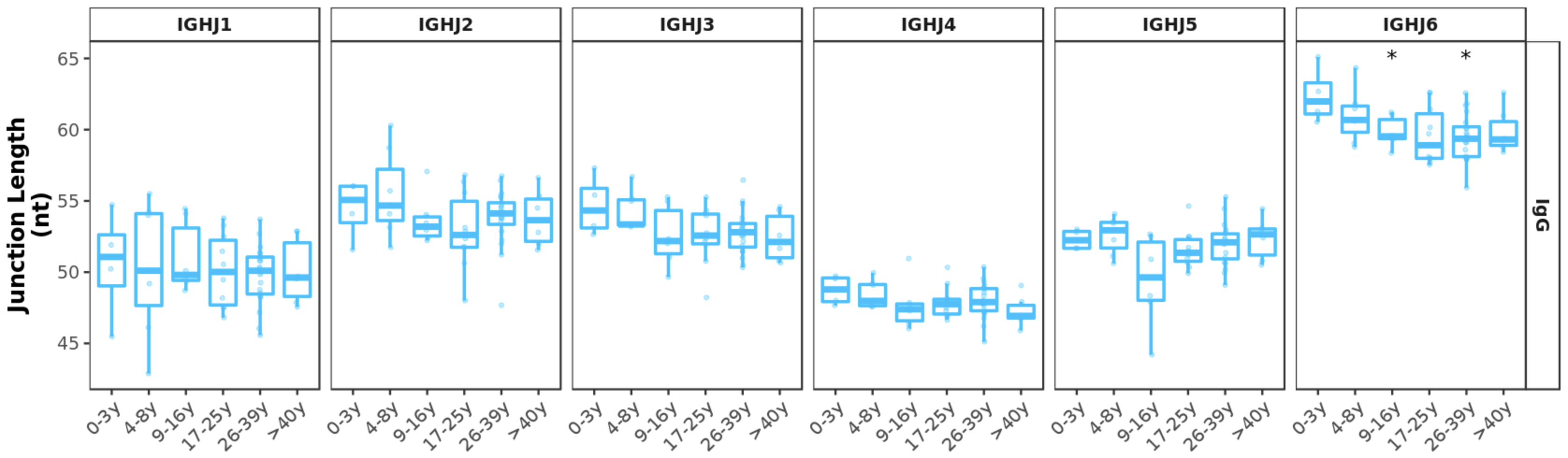


**Supplementary Figure 6:** Junction length decrease in IgG transcripts is still apparent within J gene and is only significant in transcripts with IGHJ6. Comparison of each age group to the 0-3y group was performed using the Wilcoxon test. *p<0.05

**
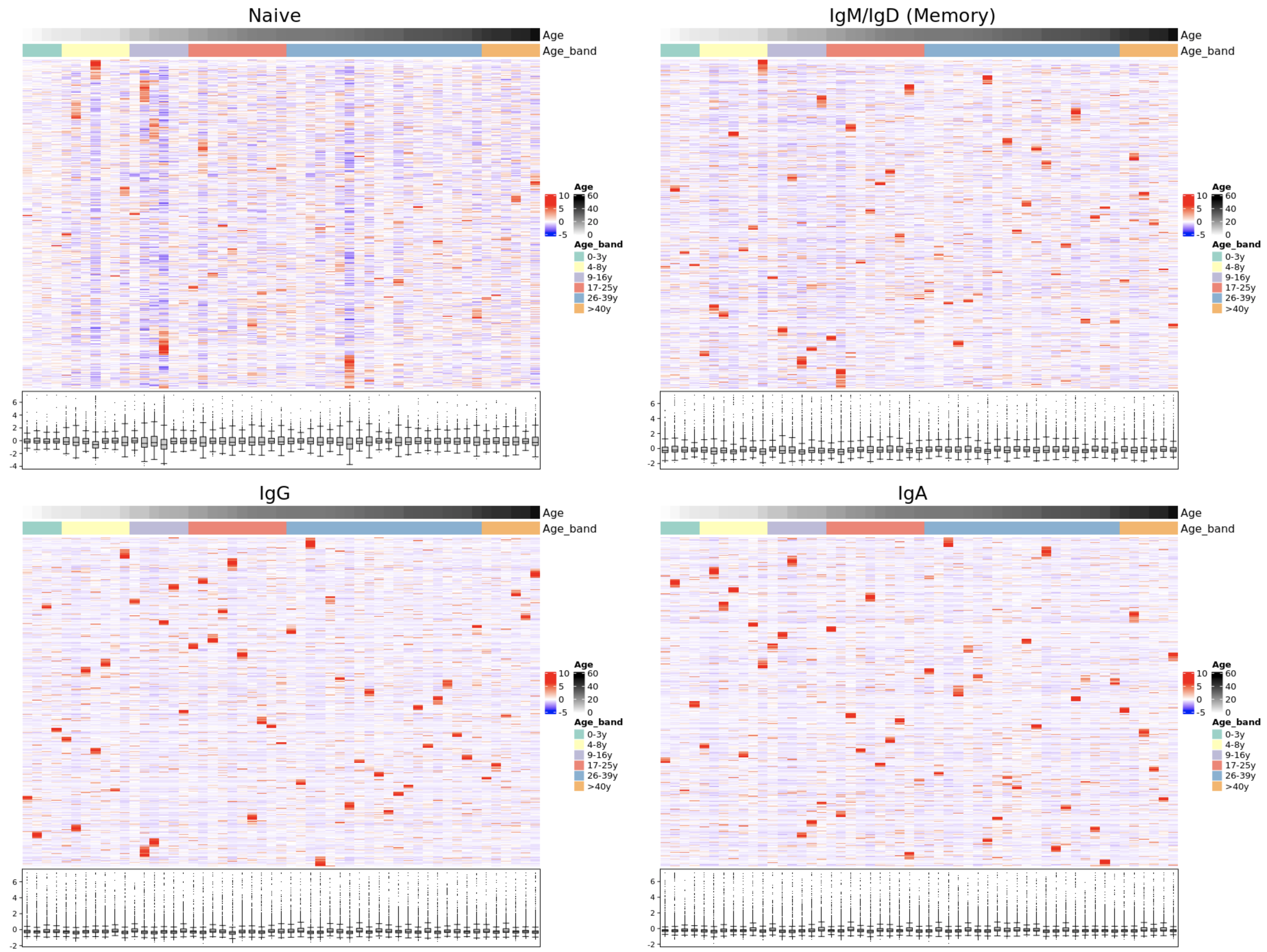
**

**Supplementary Figure 7:** The structural composition of the naïve, IgM/IgD memory and class-switched IgG and IgA repertoires. Heatmaps are normalized by row which represents usage of one PDB cluster. Distributions of the normalized PDB cluster usage by individual is shown as a boxplot. Samples (columns) are ordered by age and PDB clusters (rows) are hierarchically clustered.


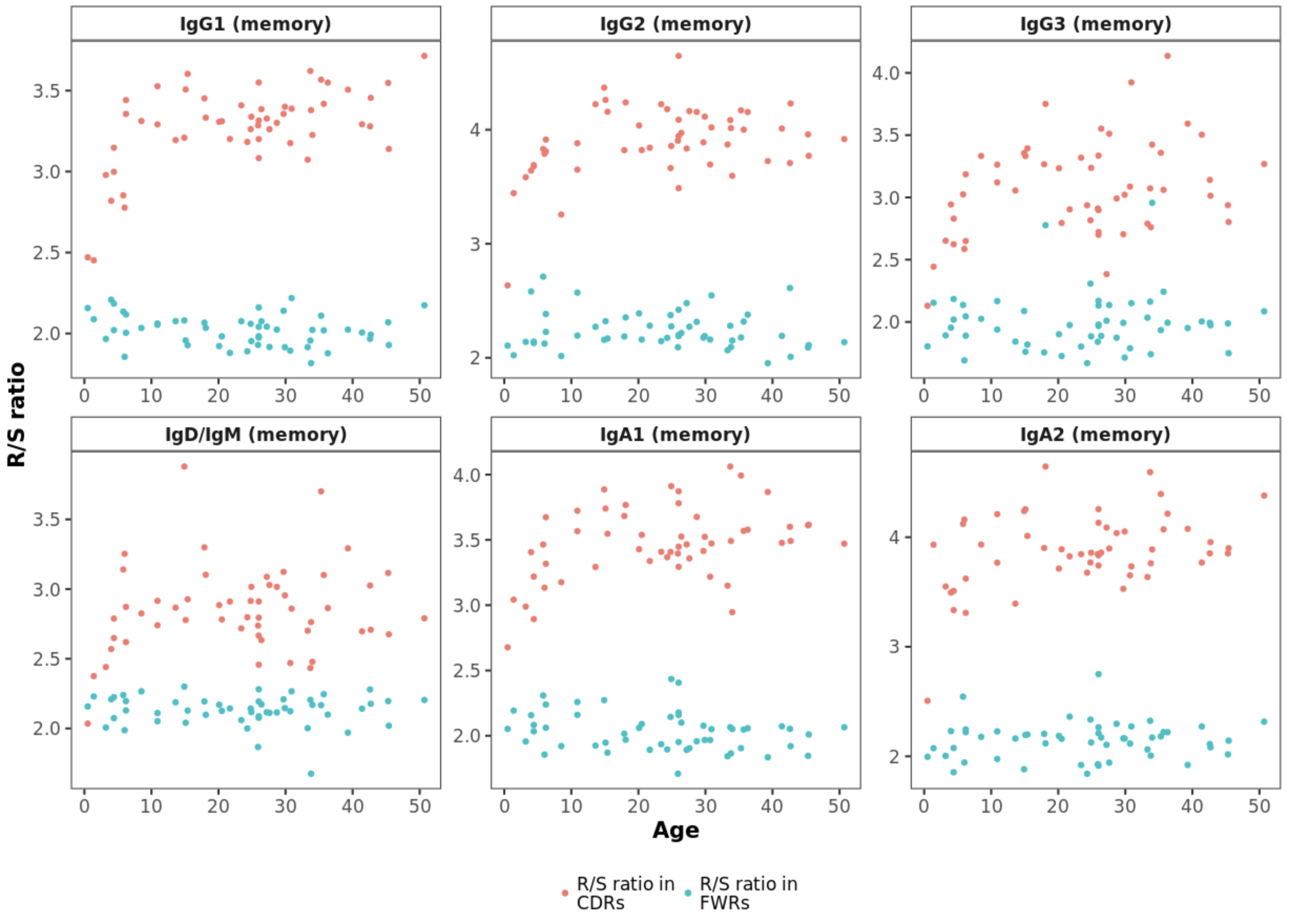


**Supplementary figure 8:** R/S ratio in FWRs does not correlate with age and is lower compared with CDRs.


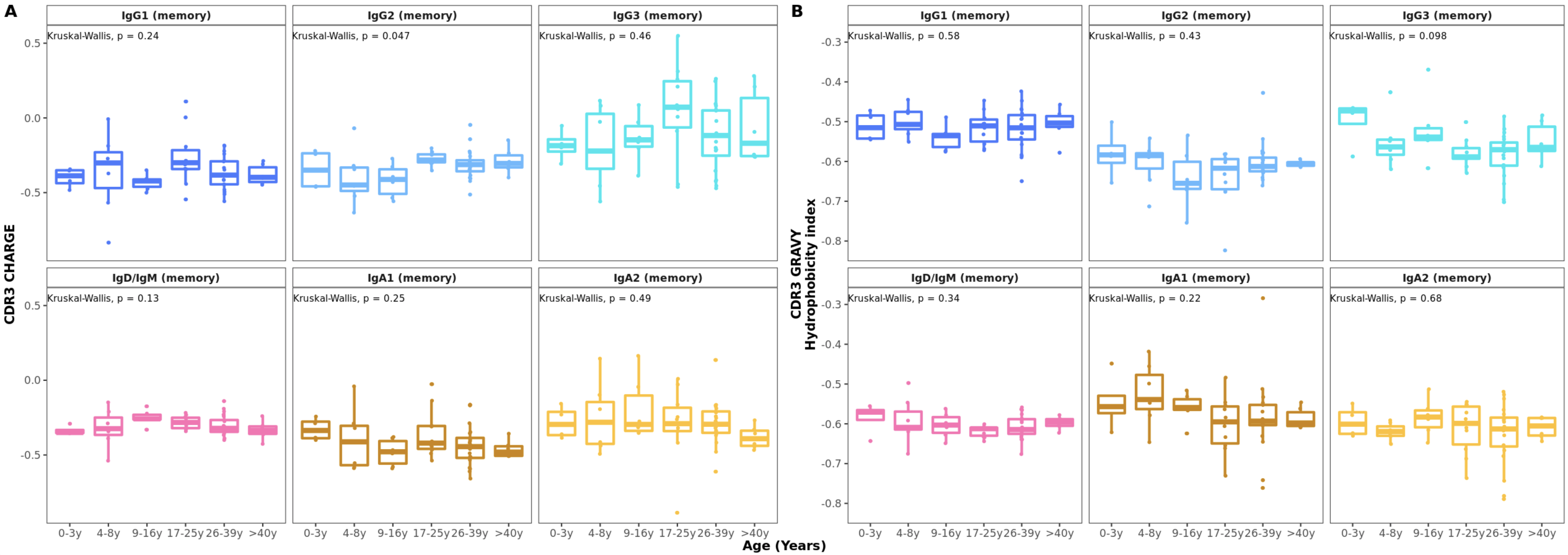


**Supplementary figure 9:** *A* CDR3 charge and *B* CDR3 hydrophobicity index do not correlate with age in healthy controls.

**Supplementary table 1**

| Participant | Sex | Age (y) | Cell type | PBMC number | B cell number | B cells estimated (E) or counted (C) | RT PCR | Raw Sequences | Unique Productive Sequences | Naive | IgD/IgM (memory) | IgG1 (memory) | IgG2 (memory) | IgG3 (memory) | IgG4 (memory) | IgA1 (memory) | IgA2 (memory) | IgE (memory) |
| --- | --- | --- | --- | --- | --- | --- | --- | --- | --- | --- | --- | --- | --- | --- | --- | --- | --- | --- |
| HC_1 | F | 0.5 | PBMCs | 14400000 | 3024000 | E | AEG,MD | 1904663 | 285839 | 231971 | 10203 | 21480 | 4276 | 4875 | 38 | 9509 | 3429 | 58 |
| HC_2 | F | 1.4 | PBMCs | 6900000 | 1932000 | E | AEG,MD | 2326250 | 239052 | 195329 | 16860 | 16237 | 1263 | 3647 | 18 | 4585 | 1111 | 2 |
| HC_3 | M | 3.2 | PBMCs | 9900000 | 2376000 | E | AEG,MD | 1622639 | 221134 | 168173 | 21993 | 11164 | 4741 | 1819 | 54 | 9894 | 3246 | 50 |
| HC_4 | F | 3.9 | PBMCs | 9450000 | 2268000 | E | AEG,MD | 1571312 | 337616 | 211936 | 49567 | 35350 | 6421 | 7459 | 46 | 22599 | 4230 | 8 |
| HC_5 | F | 4.4 | PBMCs | 4200000 | 1008000 | E | AEG,MD | 1975420 | 93299 | 65453 | 13052 | 5957 | 3788 | 1587 | 42 | 2099 | 1280 | 41 |
| HC_6 | M | 4.4 | PBMCs | 1140000 | 273600 | E | AEG,MD | 1216544 | 28674 | 23116 | 3475 | 872 | 341 | 122 | 4 | 599 | 141 | 4 |
| HC_7 | F | 5.8 | PBMCs | 11100000 | 1998000 | E | AEG,MD | 1973589 | 128326 | 79890 | 19802 | 13050 | 5047 | 1526 | 53 | 7791 | 1164 | 3 |
| HC_8 | M | 6.0 | PBMCs | 12600000 | 2268000 | E | AEG,MD | 1818496 | 124567 | 39573 | 23592 | 37668 | 5395 | 5989 | 34 | 10097 | 2147 | 72 |
| HC_9 | M | 6.2 | PBMCs | 6150000 | 1107000 | E | AEG,MD | 2474481 | 255693 | 213289 | 16278 | 5861 | 4610 | 1268 | 46 | 9844 | 4117 | 380 |
| HC_10 | F | 6.2 | PBMCs | 10800000 | 1944000 | E | AEG,MD | 1337214 | 368861 | 142685 | 61448 | 42616 | 28527 | 7659 | 534 | 59361 | 25936 | 95 |
| HC_11 | M | 8.5 | PBMCs | 10800000 | 1944000 | E | AEG,MD | 880759 | 40494 | 29337 | 4530 | 2283 | 841 | 725 | 19 | 2126 | 633 | 0 |
| HC_12 | F | 10.9 | PBMCs | 12900000 | 2064000 | E | AEG,MD | 1802230 | 366617 | 213458 | 83824 | 20972 | 10050 | 9851 | 427 | 19081 | 8746 | 208 |
| HC_13 | F | 10.9 | PBMCs | 7920000 | 1267200 | E | AEG,MD | 1168944 | 25242 | 13964 | 6767 | 1829 | 977 | 261 | 25 | 1057 | 275 | 87 |
| HC_14 | F | 13.6 | PBMCs | 4740000 | 758400 | E | AEG,MD | 872906 | 26006 | 17478 | 6899 | 278 | 644 | 70 | 5 | 450 | 173 | 9 |
| HC_15 | F | 14.9 | PBMCs | 1920000 | 307200 | E | AEG,MD | 2717449 | 44254 | 5213 | 18994 | 5991 | 3376 | 1053 | 238 | 5918 | 3464 | 7 |
| HC_16 | M | 15.1 | PBMCs | 14700000 | 2352000 | E | AEG,MD | 659493 | 95429 | 77414 | 8649 | 1635 | 2474 | 384 | 67 | 3639 | 1056 | 111 |
| HC_17 | F | 15.4 | PBMCs | NA | NA | NA | AEG,MD | 995764 | 289287 | 160495 | 29467 | 37623 | 27409 | 6542 | 1142 | 24002 | 2592 | 15 |
| HC_18 | F | 17.9 | PBMCs | 7200000 | 864000 | E | AEG,MD | 1682823 | 302272 | 95205 | 60138 | 37658 | 10783 | 12027 | 1252 | 77485 | 7704 | 20 |
| HC_19 | M | 18.1 | PBMCs | 8400000 | 1008000 | E | AEG,MD | 1011146 | 28798 | 19725 | 3478 | 1350 | 870 | 158 | 151 | 2248 | 818 | 0 |
| HC_20 | M | 20.1 | PBMCs | 13200000 | 1584000 | E | AEG,MD | 1433354 | 356105 | 146512 | 70456 | 39036 | 22756 | 4546 | 67 | 61075 | 11647 | 10 |
| HC_21 | M | 20.5 | PBMCs | 9600000 | 1152000 | E | AEG,MD | 945221 | 96859 | 54106 | 27653 | 5861 | 3223 | 381 | 684 | 3195 | 1695 | 61 |
| HC_22 | M | 21.7 | PBMCs | 15000000 | 1800000 | E | AEG,MD | 1140675 | 40591 | 30238 | 8997 | 294 | 178 | 244 | 1 | 477 | 160 | 2 |
| HC_23 | F | 23.4 | PBMCs | 9000000 | 1080000 | E | AEG,MD | 1423314 | 151168 | 93952 | 20439 | 7757 | 11844 | 1094 | 88 | 12146 | 3815 | 33 |
| HC_24 | F | 24.3 | CD19+ |  | 3600000 | C | AEG,MD | 1650432 | 53250 | 29995 | 14279 | 2320 | 2099 | 628 | 26 | 2098 | 1803 | 2 |
| HC_25 | F | 24.8 | PBMCs | 17100000 | 2052000 | E | AEG,MD | 764310 | 66786 | 36831 | 21155 | 2397 | 1562 | 308 | 0 | 3145 | 1386 | 2 |
| HC_26 | F | 24.9 | PBMCs | 18000000 | 2160000 | E | AEG,MD | 1683395 | 288827 | 126923 | 69494 | 18078 | 28519 | 2636 | 2025 | 26499 | 14562 | 91 |
| HC_27 | M | 25.9 | PBMCs | 12000000 | 1440000 | E | Mix I | 593323 | 52933 | 34902 | 12049 | 2280 | 2011 | 432 | 17 | 509 | 728 | 5 |
| HC_28 | F | 26.0 | PBMCs | 14000000 | 1680000 | E | Mix I | 1146458 | 133861 | 75380 | 31145 | 10845 | 10135 | 1339 | 123 | 2477 | 2399 | 18 |
| HC_29 | F | 26.0 | CD19+ |  | 2000000 | C | AEG,MD | 1710083 | 235120 | 175862 | 42382 | 5427 | 3685 | 1335 | 17 | 4627 | 1783 | 2 |
| HC_30 | F | 26.0 | PBMCs | 3300000 | 185000 | C | AEG,MD | 2347978 | 158947 | 112391 | 27918 | 6055 | 4737 | 1279 | 99 | 5434 | 1033 | 1 |
| HC_31 | F | 26.0 | PBMCs | 1725000 | 68000 | C | AEG,MD | 1524836 | 99870 | 54673 | 29814 | 4476 | 3962 | 708 | 102 | 4847 | 1288 | 0 |
| HC_32 | F | 26.4 | PBMCs | 14100000 | 1692000 | E | AEG,MD | 1581380 | 168295 | 107868 | 15198 | 12723 | 6925 | 6070 | 230 | 13898 | 5288 | 95 |
| HC_33 | F | 27.2 | PBMCs | 13000000 | 1560000 | E | Mix I | 1217105 | 71742 | 37514 | 24133 | 4257 | 2197 | 1173 | 36 | 1479 | 953 | 0 |
| HC_34 | F | 27.6 | CD19+ | 11250000 | 240000 | C | AEG,MD | 941177 | 31596 | 9300 | 14638 | 1496 | 2727 | 208 | 52 | 2155 | 1020 | 0 |
| HC_35 | F | 28.7 | PBMCs | 8700000 | 1044000 | E | AEG,MD | 1824932 | 176403 | 84366 | 52562 | 11813 | 8105 | 1447 | 249 | 11565 | 6271 | 25 |
| HC_36 | F | 29.7 | PBMCs | 8100000 | 972000 | E | AEG,MD | 1103351 | 45312 | 24574 | 11748 | 2324 | 1406 | 233 | 10 | 3726 | 1291 | 0 |
| HC_37 | M | 29.9 | PBMCs | 12000000 | 1440000 | E | AEG,MD | 1909727 | 338646 | 184195 | 99152 | 11746 | 23501 | 1585 | 425 | 8537 | 9479 | 26 |
| HC_38 | F | 30.7 | PBMCs | 12750000 | 1530000 | E | AEG,MD | 1877484 | 366879 | 255140 | 27649 | 13883 | 27944 | 3806 | 323 | 24438 | 13686 | 10 |
| HC_39 | M | 30.9 | PBMCs | 12600000 | 1512000 | E | AEG,MD | 640010 | 80488 | 30441 | 15974 | 3007 | 11972 | 658 | 5 | 8369 | 10062 | 0 |
| HC_40 | M | 33.3 | CD19+ | 31800000 | 680000 | C | Mix I | 437659 | 55698 | 41275 | 8336 | 2617 | 1390 | 993 | 55 | 811 | 220 | 1 |
| HC_41 | F | 33.7 | PBMCs | 69000000 | 1380000 | E | AEG,MD | 1786984 | 132252 | 101056 | 17389 | 4499 | 2946 | 595 | 52 | 4578 | 1134 | 3 |
| HC_42 | F | 33.8 | CD19+ | 100000000 | 3360000 | C | AEG,MD | 1032835 | 195330 | 149483 | 26300 | 6294 | 3445 | 1021 | 139 | 4069 | 4566 | 13 |
| HC_43 | F | 34.0 | PBMCs | 3000000 | 123000 | C | AEG,MD | 1598313 | 97235 | 81449 | 8699 | 2844 | 1067 | 336 | 49 | 2136 | 636 | 19 |
| HC_44 | M | 35.3 | PBMCs | 15000000 | 1800000 | E | Mix I | 4584690 | 303597 | 130253 | 129632 | 19339 | 7425 | 3176 | 94 | 7750 | 5926 | 2 |
| HC_45 | M | 35.7 | PBMCs | 6480000 | 777600 | E | AEG,MD | 943459 | 124374 | 60998 | 50902 | 3062 | 4235 | 701 | 37 | 3080 | 1359 | 0 |
| HC_46 | F | 36.3 | PBMCs | 21600000 | 1300000 | E | AEG,MD | 1833493 | 187445 | 121444 | 25433 | 7927 | 16069 | 982 | 224 | 10156 | 5193 | 17 |
| HC_47 | M | 39.3 | PBMCs | 51000000 | 6120000 | E | Mix I | 454024 | 47055 | 23248 | 15966 | 3212 | 1697 | 701 | 17 | 1230 | 968 | 16 |
| HC_48 | M | 41.4 | PBMCs | 16500000 | 1980000 | E | AEG,MD | 903117 | 110233 | 65831 | 30775 | 2524 | 6928 | 640 | 5 | 2326 | 1204 | 0 |
| HC_49 | M | 42.6 | PBMCs | 5700000 | 684000 | E | AEG,MD | 878951 | 86541 | 69361 | 9171 | 2296 | 2206 | 360 | 49 | 2121 | 947 | 30 |
| HC_50 | M | 42.7 | PBMCs | 10000000 | 1200000 | E | AEG,MD | 1668134 | 69380 | 37942 | 9587 | 3172 | 3722 | 1135 | 78 | 9926 | 3805 | 13 |
| HC_51 | F | 45.3 | PBMCs | 8400000 | 1008000 | E | AEG,MD | 1445580 | 168554 | 54396 | 50347 | 9925 | 27511 | 1502 | 143 | 16832 | 7879 | 19 |
| HC_52 | F | 45.4 | PBMCs | 13200000 | 1580000 | E | AEG,MD | 1694914 | 261324 | 166520 | 53179 | 9798 | 8977 | 4003 | 316 | 11782 | 6690 | 59 |
| HC_53 | F | 50.7 | PBMCs | 9600000 | 1152000 | E | AEG,MD | 1970119 | 116853 | 41529 | 40864 | 15563 | 4098 | 1241 | 47 | 8088 | 5414 | 9 |

**Supplementary table 2**

| **Primer** | **Sequence** |
| --- | --- |
| CHA_70x | GTGACTGGAGTTCAGACGTGTGCTCTTCCGATCTNNNNTNNNNTNNNNGAYGACCACGTTCCCATC*T |
| CHM_70x | GTGACTGGAGTTCAGACGTGTGCTCTTCCGATCTNNNNTNNNNTNNNNTCGTATCCGACGGGGAATT*C |
| CHD_70x | GTGACTGGAGTTCAGACGTGTGCTCTTCCGATCTNNNNTNNNNTNNNNGGGCTGTTATCCTTTGGGT*G |
| CHE_70x | GTGACTGGAGTTCAGACGTGTGCTCTTCCGATCTNNNNTNNNNTNNNNAGAGTCACGGAGGTGGCAT*T |
| CHG_70x | GTGACTGGAGTTCAGACGTGTGCTCTTCCGATCTNNNNTNNNNTNNNNAGTAGTCCTTGACCAGGCA*G |
| IGHV1_50x | ACACTCTTTCCCTACACGACGCTCTTCCGATCTGGCCTCAGTGAAGGTCTCCTGCAA*G |
| IGHV2_50x | ACACTCTTTCCCTACACGACGCTCTTCCGATCTGTCTGGTCCTACGCTGGTGAAACC*C |
| IGHV3_50x | ACACTCTTTCCCTACACGACGCTCTTCCGATCTCTGGGGGGTCCCTGAGACTCTCCT*G |
| IGHV4_50x | ACACTCTTTCCCTACACGACGCTCTTCCGATCTCTTCGGAGACCCTGTCCCTCACCT*G |
| IGHV5_50x | ACACTCTTTCCCTACACGACGCTCTTCCGATCTCGGGGAGTCTCTGAACATCTCCTG*T |
| IGHV6_50x | ACACTCTTTCCCTACACGACGCTCTTCCGATCTTCGCAGACCCTCTCACTCACCTGT*G |
| PCR1_rev | CTAGCCTTCTCGTGTGCAGACTTGAGGTCAGTG |
| AD50x_i1 | AATGATACGGCGACCACCGAGATCTACACTATAGCCTACACTCTTTCCCTACACGACGCTCTTCCGATC*T |
| AD50x_i2 | AATGATACGGCGACCACCGAGATCTACACATAGAGGCACACTCTTTCCCTACACGACGCTCTTCCGATC*T |
| AD50x_i3 | AATGATACGGCGACCACCGAGATCTACACCCTATCCTACACTCTTTCCCTACACGACGCTCTTCCGATC*T |
| AD50x_i4 | AATGATACGGCGACCACCGAGATCTACACGGCTCTGAACACTCTTTCCCTACACGACGCTCTTCCGATC*T |
| AD50x_i5 | AATGATACGGCGACCACCGAGATCTACACAGGCGAAGACACTCTTTCCCTACACGACGCTCTTCCGATC*T |
| AD50x_i6 | AATGATACGGCGACCACCGAGATCTACACTAATCTTAACACTCTTTCCCTACACGACGCTCTTCCGATC*T |
| AD50x_i7 | AATGATACGGCGACCACCGAGATCTACACCAGGACGTACACTCTTTCCCTACACGACGCTCTTCCGATC*T |
| AD50x_i8 | AATGATACGGCGACCACCGAGATCTACACGTACTGACACACTCTTTCCCTACACGACGCTCTTCCGATC*T |
| AD70x_i1 | CAAGCAGAAGACGGCATACGAGATCGAGTAATGTGACTGGAGTTCAGACGTGTGCTCTTCCGAT*C |
| AD70x_i2 | CAAGCAGAAGACGGCATACGAGATTCTCCGGAGTGACTGGAGTTCAGACGTGTGCTCTTCCGAT*C |
| AD70x_i3 | CAAGCAGAAGACGGCATACGAGATAATGAGCGGTGACTGGAGTTCAGACGTGTGCTCTTCCGAT*C |
| AD70x_i4 | CAAGCAGAAGACGGCATACGAGATGGAATCTCGTGACTGGAGTTCAGACGTGTGCTCTTCCGAT*C |
| AD70x_i5 | CAAGCAGAAGACGGCATACGAGATTTCTGAATGTGACTGGAGTTCAGACGTGTGCTCTTCCGAT*C |
| AD70x_i6 | CAAGCAGAAGACGGCATACGAGATACGAATTCGTGACTGGAGTTCAGACGTGTGCTCTTCCGAT*C |
| AD70x_i7 | CAAGCAGAAGACGGCATACGAGATAGCTTCAGGTGACTGGAGTTCAGACGTGTGCTCTTCCGAT*C |
| AD70x_i8 | CAAGCAGAAGACGGCATACGAGATGCGCATTAGTGACTGGAGTTCAGACGTGTGCTCTTCCGAT*C |
| AD70x_i9 | CAAGCAGAAGACGGCATACGAGATCATAGCCGGTGACTGGAGTTCAGACGTGTGCTCTTCCGAT*C |
| AD70x_i10 | CAAGCAGAAGACGGCATACGAGATTTCGCGGAGTGACTGGAGTTCAGACGTGTGCTCTTCCGAT*C |
| AD70x_i11 | CAAGCAGAAGACGGCATACGAGATGCGCGAGAGTGACTGGAGTTCAGACGTGTGCTCTTCCGAT*C |
| AD70x_i12 | CAAGCAGAAGACGGCATACGAGATCTATCGCTGTGACTGGAGTTCAGACGTGTGCTCTTCCGAT*C |

| **Reverse Transcription** |  |  | |  | |  | |  | |  | |  | |  | |  |  | |  |
| --- | --- | --- | --- | --- | --- | --- | --- | --- | --- | --- | --- | --- | --- | --- | --- | --- | --- | --- | --- |
| Primers_mix | MD mix | | | AEG mix | | | | | | Mix I | | | | | | | | |  |
| Primers | CHM_70x | CHD_70x | | CHA_70x | | CHE_70x | | CHG_70x | | CHM_70x | | CHD_70x | | CHA_70x | | CHE_70x | CHG_70x | | Total |
| Concentration (uM) | 0.625 | 0.625 | | 0.25 | | 0.25 | | 0.75 | | 0.03125 | | 0.03125 | | 0.0625 | | 0.125 | 1 | | 1.25 |
| **PCR1** |  | |  | |  | |  | |  | |  | |  | |  | | |  |  |
| Primers | IGHV1_50x | | IGHV2_50x | | IGHV3_50x | | IGHV4_50x | | IGHV5_50x | | IGHV6_50x | | PCR1_rev | | Total | | |  |  |
| Concentration (uM) | 1.25 | | | | | | | | | | | | 1.25 | | 2.5 | | |  |  |
|  |  | |  | |  | |  | |  | |  | |  | |  | | |  |  |
| **PCR2** |  | |  | |  | |  | |  | |  | |  | |  | | |  |  |
| Primer | AD70x | | AD50x | | Total | |  | |  | |  | |  | |  | | |  |  |
| Concentration (uM) | 1.25 | | 1.25 | | 2.5 | |  | |  | |  | |  | |  | | |  |  |

**Supplementary table 3**

| **Age band** | **Number of individuals** | **Minimum age** | **Maximum Age** | **Mean number of raw sequences** | **Mean number of unique sequences** |
| --- | --- | --- | --- | --- | --- |
| 0-3y | 4 | 0.5 | 3.9 | 1856216 | 270910 |
| 4-8y | 7 | 4.4 | 8.5 | 1668071 | 148559 |
| 9-16y | 6 | 10.9 | 15.4 | 1369464 | 141139 |
| 17-25y | 10 | 16.9 | 25.9 | 1232799 | 143759 |
| 26-39y | 20 | 26 | 39.3 | 1524799 | 152507 |
| >40y | 6 | 41.4 | 50.7 | 1426803 | 135480 |
